# The Effect of Composol Medium on miR-16 Expression during Platelet Storage up to Day 7 at Room Temperature

**DOI:** 10.22074/cellj.2021.6790

**Published:** 2020-04-22

**Authors:** Ali Rajabi, Zohreh Sharifi, Fatemeh Yari, Mohammadreza Deyhim, Mohammadali Jalili

**Affiliations:** Blood Transfusion Research Center, High Institute for Research and Education in Transfusion Medicine, Tehran, Iran

**Keywords:** Blood Platelets, MicroRNAs, miR-16, Platelet Storage

## Abstract

**Objective:**

MicroRNAs (miRNAs) are short, noncoding RNAs that play vital roles in gene regulation. It has been shown that storage has an effect on platelet miRNAs. MiR-16 is highly expressed in platelets and it appears to target the genes involved in cell death. It has been shown that platelets could be stored in Composol for a longer period of time. The aim of the present study was to assess and compare the expression pattern of miR-16 in platelet concentrates (PCs) in plasma and Composol media.

**Materials and Methods:**

In an experimental study, ten PC bags were collected and each bag was divided into two separate bags, one with the 70% Composol and the other with only plasma. Both bags were stored for 7 days at 22˚C and tested on days 1, 3, 5, and 7 of storage. For each sample, we performed quantitative real-time polymerase chain reaction (qRT-PCR). The water-soluble tetrazolium salt-1 (WST-1) test was used to assess platelet viability in all of the samples. Statistical analysis was done by SPSS and REST software. A P<0.05 was considered statistically significant.

**Results:**

miR-16 was significantly elevated during the storage days, with fold changes of 3.47 (plasma) and 2.77 (Composol). The Composol group had significantly decreased miR-16 expression compared with the plasma group. Results of the WST-1 test showed less decrease in optical density (OD) in the Composol group (0.93 ± 0.4) during the storage days compared with the plasma group (0.75 ± 0.3).

**Conclusion:**

Our finding supported results from previous studies that reported an increase in miR-16 expression during platelet storage. In addition, miR-16 down-regulation in Composol medium implied that Composol might be a good solution for long-term platelet storage because it has the potential to elevate the shelf-life of platelets stored at 22˚C.

## Introduction

MicroRNAs (miRNAs) are short (20-24 nucleotides), single-strand, noncoding evolutionarily conserved RNAs that mediate post-transcriptional negative regulation of gene expression by recognizing and binding mRNA transcripts (1-3). They were first discovered in the nematode *Caenorhabditis elegans* in 1993 (4). MiRNAs appear to target several mRNAs (4, 5), an average of 307 distinct mRNAs for one particular miRNA (6). More than 60% of mammalian mRNAs are affected by miRNAs (7). They are considered to be negative regulators because they bind to mRNA targets and then silence their translation (8). To date, the number of discovered miRNAs is more than 2000 and they have been investigated more than all other non-coding RNAs (9, 10).

Platelets express high levels of various miRNAs that have been derived from their precursor cells, megakaryocytes (11, 12). There is a lack of knowledge about platelet miRNAs’ behaviour under storage conditions (13). It has been shown that storage time has an effect on platelet miRNAs. Some miRNAs have different patterns of expression during storage (14, 15). miRNA-16 (miR-16) appears to have an increased level tendency during platelet storage (15, 16). It was first discovered through profile expression analysis of chronic lymphocytic leukaemia patients. miR-16 binds to and targets a nine base pair sequence in the 3’-UTR region of the anti-apoptotic gene *BCL-2*, which is a crucial gene in programmed cell death. miR-16 negatively regulates *BCL-2* at the posttranscriptional level (17). It is assumed that platelet cell death is mediated by miRNAs, and miR- 16 is an apoptotic factor for accelerating cell death.

Platelet additive solutions (PASs) are useful solutions added to platelet concentrates (PCs) to make the cells more viable over longer periods of time. PASs prevent the PC recipients from increased plasma exposure and provide lower risk of transfusion reactions. In these cases, the plasma content of PCs is replaced by PASs. PCs could be stored more than five days under good storage situations that prevent bacterial contamination and with the use of additive solutions (18). There are many PASs that have been introduced, each of which has a different composition that emphasizes multiple aspects of platelet needs. The Composol solution (known as PAS-D) is a third generation PAS salt composed of sodium chloride, sodium gluconate, sodium acetate trihydrase, sodium citrate dehydrate, potassium chloride, magnesium chloride hexahydrate, HCl, and water. The ingredients, especially magnesium, calcium, potassium and citrate have good effects on platelet membrane function, rate of glycolysis and platelet activation. According to multiple reports, Composol is a potent PAS for long-term PC storage. PCs stored in Composol medium have shown better function with less activated platelets (19-23).

No study has compared platelet miRNAs expression in different PAS media until now. The aim of this study was to investigate platelet miR-16 expression during PC storage periods and the effect of Composol on miR-16 expression.

## Materials and Methods

### Platelet concentrate collection and sample preparation

This experimental study was approved by the Research Ethics Committee of the High Institute for Research and Education in Transfusion Medicine under the code IR.TMI.REC.1395.010.

Ten single donor PC bags from healthy volunteers were prepared from whole blood bags by the platelet rich plasma method in the Iranian Blood Transfusion Organization. All platelet bags (Macopharma, France) were counted for platelet numbers by a Sysmex K-1000 Hematolgy Analyzer (Sysmex, Japan). Then, the content of each bag was divided equally into two separate bags using a transfer bag and a connective device, CompoDock instrument (Fresenius, Germany). The two bags were separated by a thermic tube sealer device (Fresenius, Germany) and both bags were centrifuged at 5000 g for 6 minutes at 22˚C in a blood bag centrifuge (model 830RS, Hetich, Germany). After removing 70% of the plasma from one of the bags by using a manual plasma extractor, we carefully added Composol solution (Composol-PS, E2083, Fresenius, Germany) and used this bag as the test (Composol) group. We followed aseptic techniques when adding the Composol to the PC bags. The other intact bag contained only plasma and was considered to be the control (plasma) group.

Both the plasma and Composol PC bags were stored with constant agitation on a PC shaker-incubator (Model 48PIAG-93-A, Fajr DP, Iran) at 20-24˚C for seven days. We simultaneously tested both PC groups on storage days 1, 3, 5, and 7. Day zero was the processing day or the day before the onset of testing. Platelet counts and volume of both the plasma and Composol samples were unified. We used the same volume and concentration of platelets for analyses on each of the test days. Less than 24 hours after sample processing, the tests were started with fresh platelets.

In order to detect bacterial contamination, all PC bags were sampled on the first (day 1) and last (day 7) storage day points and cultured on general blood agar and eosin methylene blue (EMB) agar media (Merck, Germany). For minimizing the effect of nucleated cells in molecular testing, both plasma- and Composol-PC tubes were centrifuged at 96 g for 4 minutes in a bench top centrifuge (Sigma, Germany) to reduce the white blood cell numbers.

### MicroRNA extraction and analysis by real-time polymerase chain reaction

miRNA was extracted using a SanPrep column microRNA Mini-Prep kit (Bio Basic, Inc., Canada) according to the manufacturer’s instructions. mRNA polyadenylation and cDNA synthesis were assessed with BONmiR qPCR kits (Stem Cell Technology, Iran).

Standard curves of miR-16 and U6 snRNA (*RNU6*) were plotted using serial dilutions of the cDNAs to evaluate the quantitative real-time polymerase chain reaction (qRT-PCR) efficiency. qRT-PCR with R2=0.998 and curve slope=3.358 was performed for all samples using a Rotor-Gene Q cycler (Qiagen, Germany) according to the manufacturer’s guidelines under the following conditions: 95˚C for 2 minutes (one cycle), 95˚C for 5 seconds and 60˚C for 30 seconds (40 cycles). Melting curve analysis was done by heating from 50˚C to 95˚C at a rate of 0.1˚C/second. The PCR primer for the standard sequence of miR-16-5p according to the miRBase database (mirbase.org) was used as the specific forward primer (5´-GGCATAGCAGCACGTAAAT-3´) in conjunction with the *RNU6* gene forward primer (5´-AACGATACAGAGAAGATTAG-3´) as the internal control and reference housekeeping gene. A common reverse primer was also used for the reactions.

Results were taken as cycles of threshold (CTs) and relative gene expression was obtained using the standard comparative CT (∆∆CT) method (24). All samples were run in triplicate and the mean CT values were used as the raw and primary results. For relative gene expression and fold change analysis, we used REST software (REST- 2009©, rest.gene-quantification.info). *RNU6* CT results were used for gene expression normalization.

### Platelet viability assay

The water-soluble tetrazolium salt-1 (WST-1) test was used to assess platelet viability in the two groups with the WST-1 cell proliferation assay kit (Cayman Chemical, Ann Arbor, MI, USA) and a 96-well microplate. All PC samples were centrifuged at 1800 g for 4 minutes and the platelets were re-suspended in phosphate-buffered saline. Platelet concentrations of 5×10^11^ cells/L in a 100 μL suspension were used with the addition of 10 μL of WST-1 reagent mix, followed by incubation for 4 hours in a CO_2_ incubator at 37˚C. The absorbance of the reaction was read at 450 nm as the optical density (OD) in a microplate reader (ASYS Expert 96 UV Microplate Reader, UK). Results of the WST-1 analysis were presented as mean ± SD.

### Statistical analysis

The data were analysed using IBM SPSS Statistics version 23 (IBM, USA). To compare the results of two PC groups on corresponding days, we performed the paired t test. Analysis of variance (ANOVA) for repeated measures was done to assess the differences at various storage times. A P<0.05 was considered to indicate a statistically significant difference.

## Results

### Platelet count and microbial analysis

The untreated plasma group samples contained more than 1×10^12^ platelets/L on the first day of storage. The mean ± SD platelet counts on the first day were 1.15 ± 0.10×10^12 ^platelets/L. Upper and lower limits were 1.35×10^12^ platelets/L and 1.03×10^12^ platelets/L, respectively. The Composol bags had slightly less platelet counts than their primary plasma bags because of the centrifugation process. The mean ± SD platelet counts in the Composol group on the first storage day were 0.99 ± 0.08×10^12^ platelets/L, and the upper and lower limits were 1.15×10^12^ platelets/L and 0.82×10^12^ platelets/L, respectively. However, the count was the same in both paired samples before the start of each testing day. Microbial analyses were conducted on the first and last days of storage before the start of qRTPCR testing. The analysis of results showed no microbial contamination in any of the samples.

### Quantitative real-time polymerase chain reaction results

**Fig.1:**
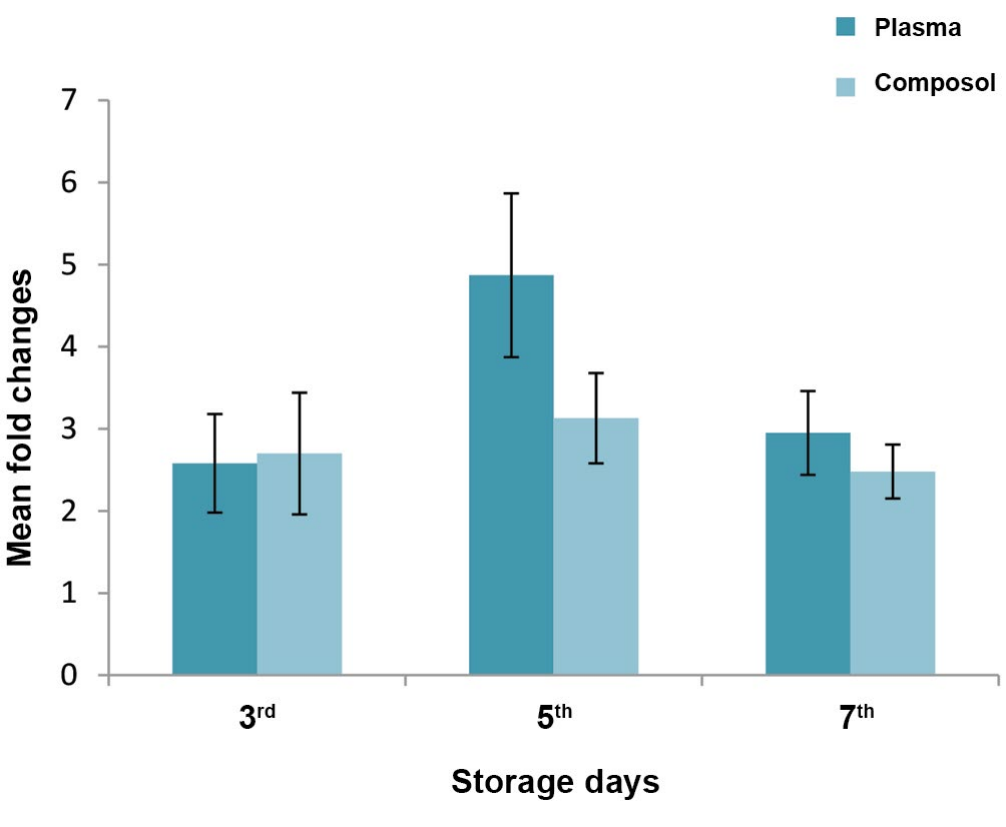
Mean fold changes of miR-16 during the storage days in both platelet concentrates (PCs) groups. Days 3, 5 and 7 were compared to day 1 of storage. Amounts were calculated by REST software, where the raw CT results were converted to fold changes. Fold changes imply the amount of changes in comparison to the baseline level of gene expression.

The qRT-PCR results originally are expressed as raw CTs. All samples were run in triplicate and simultaneously with the control gene*, RNU6*. For reporting the results, we calculated the raw CTs into simple fold changes by REST software to show the amount of *miR-16* gene expression. We found that *miR-16* expression was elevated during the storage days in both the plasma and Composol groups compared with the *RNU6* internal control. Day to day comparison of both group samples against the first storage day (day 1) showed a clear increase in *miR-16* expression during storage. This increase was significant for days 3 and 5 in both groups, but not for day 7 when compared with day 1 (Table 1, Fig.1).

**Table 1:** Expression of miR-16 in the plasma and Composol groups


Storage days versus storage day 1	Plasma		Composol	
	Mean fold change	P value	Mean fold change	P value

3	2.58	0.027	2.70	0.029
5	4.87	0.037	3.13	0.048
7	2.95	0.124	2.48	0.115


Storage days 3, 5 and 7 were compared to the first storage day. P values were calculated by the t test for paired samples. A P<0.05 indicated a statistically significant difference.

### Fold change analysis

The average fold changes in miR-16 expression in all days in comparison with the first storage day were 3.5 in plasma and 2.8 in Composol group. The most statistically significant increase in miR-16 was seen on day 5 of storage in both PC groups. A fold change of 4.87 (P=0.037) was seen in the plasma group and a fold change of 3.13 (P=0.048) was observed in the Composol group (Table 1).

Despite the obvious increased expression of miR-16 in both PC groups for all storage days after the third day, miR- 16 expression decreased in the Composol group compared with the plasma group (average fold change of 0.356). This expression decrease for all days was statistically significant, except for day 7 of storage (Table 2).

**Table 2:** Comparison of miR-16 expression in the Composol versus plasma platelet concentrate (PC) group


Storage days	Mean fold change	P value

1	0.382	0.049
3	0.399	0.015
5	0.277	0.045
7	0.365	0.102


P values were calculated by the t test for paired samples. P<0.05 was considered statistically significant.

### Viability assessment

**Fig.2:**
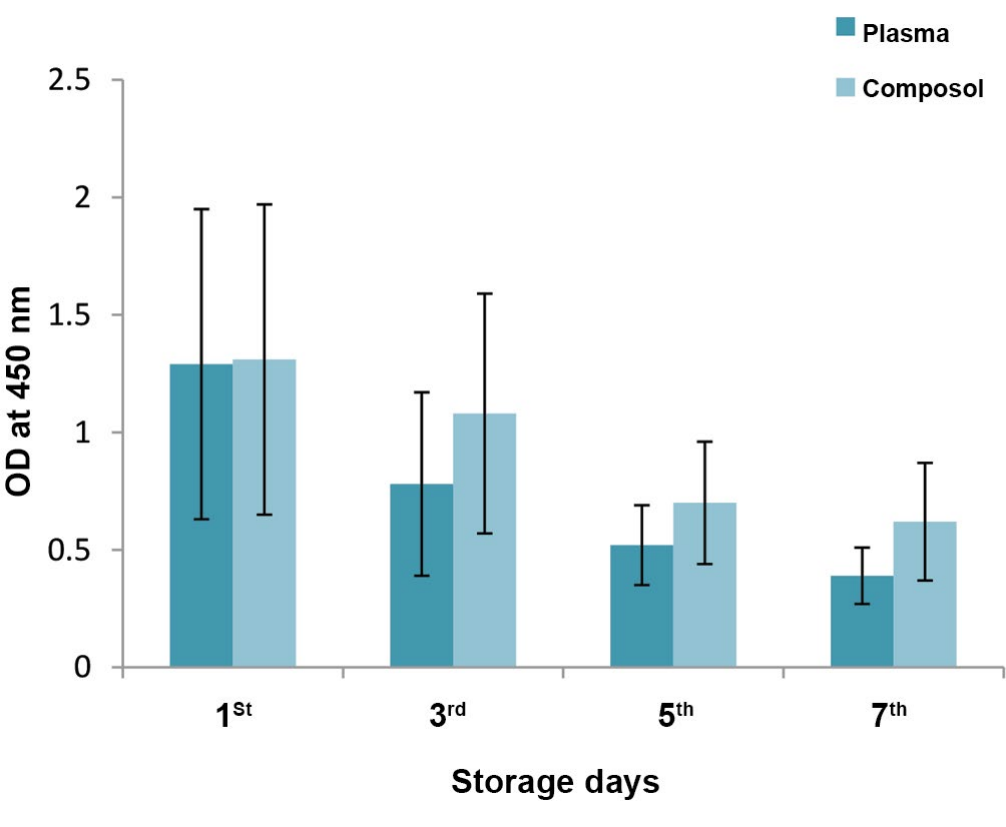
WST-1 assay test with one standard deviation (1SD) in different storage days as optical density (OD) of WST-1 samples at 450 nm. Corresponding days in both group samples were compared and P values were calculated by the t test for paired samples. P<0.05 was considered statistically significant.

Results of the WST-1 test for platelet viability revealed that the ODs of WST-1 gradually fell during storage in both groups. This decrease was more obvious from day 3 of storage (Fig.2). We also showed that platelets were more viable in the Composol samples in comparison with the control plasma samples. The plasma group had an average OD of 0.75 ± 0.3, whereas the Composol group had an average OD of 0.93 ± 0.4 (Table 3, Fig.2).

**Table 3:** Results of WST-1 test for the Composol and plasma groups as mean ± SD of the optical density


Storage day	Optical density		P value
	Plasma	Composol	

1	1.29 ± 0.56	1.31 ± 0.66	0.493
3	0.780 ± 0.39	1.08 ± 0.51	0.034
5	0.521 ± 0.17	0.703 ± 0.26	0.049
7	0.394 ± 0.17	0.620 ± 0.25	0.067


Optical density were read at 450 nm. P values were calculated by the t test for paired samples. P<0.05 were considered significant. Comparisons were done between plasma (control) and Composol (test) groups for each of the corresponding storage days.

## Discussion

Platelets play very important roles for normal blood haemostasis and effective coagulation function at times of injury. They preserve vascular integrity (25). PCs are used to control bleeding in patients with low platelet counts or impaired platelet functionality. Because of the low (3-5 days) shelf-life of platelets on storage conditions, they are valuable blood components that should be kept for destitute patients.

Platelets lack nucleus, but they have mRNA synthesis (26). miRNAs are involved in fine-tuning control of gene expression (27, 28). They suppress mRNAs by inhibiting translation (29). An attractive issue is that they can put on their regulation in the storage conditions (13). miRNAs have vital roles in essential cellular functions, including cell apoptosis (30, 31). Platelets have several miRNAs and, to date, many studies have researched miRNAs in platelets, each considered one or more aspects of the platelet characteristics, namely their roles in diseases, blood banks, etc.

In this study, we found that miR-16 expression in both the plasma and Composol groups increased during PC storage. The most obvious increase was found on day 5 of storage, with mean fold changes of 4.87 (plasma) and 3.13 (Composol). However, the average increased expression was less in the Composol group (2.8) compared to the plasma group (3.5). To date, no reports have compared miRNA expression in plasma and PAS medium. In 2015, Pontes et al. (14) analysed 16 PC bags in an attempt to characterize the expression profile of platelet miRNAs. They found a total of 1899 miRNAs over six selected storage days and listed the most highly expressed miRNAs in each storage day (until day 7). No information about miR-16 was mentioned in their report. Maués et al. (32) examined the expression profiles of miRNAs from 100 PCs stored for six days at room temperature. They found that nine miRNAs had down-regulated and five had upregulated profiles. They did not conduct any experiment with miR-16. Kannan et al. (16) reported a change in different apoptosis-associated miRNA levels during storage as assessed by miRNA array. They observed that some of the miRNAs increased, including miR-16 and Let-7b, during storage. In 2014, Yu et al. (15) reported the expression patterns of some apoptosis-associated miRNAs in apheresis platelets. In their study, five miRNA were up-regulated, including miR-16, and five were downregulated. We also reported the increased expression of miR-16 in all PC bags during the assessed storage days, which supported the results of the above reports.

miR-16 is a marker of apoptosis. It is best known for its role in haematological and non-haematological diseases such as leukaemia, diabetes and solid tumours (17, 33- 37), because of the role of platelets in inflammation and other biological processes. The important role of miR-16 in platelet gene regulation has been proven. Therefore, a decreased level of miR-16 is associated with more viable platelets in the PC bags. For this reason, miR- 16 is a good predictor of platelet viability in PC bags stored under normal storage conditions. More than 1000 targets have been identified for miR-16, including platelet lipoxygenase and CD151 antigen, which are specific for platelet apoptosis (16).

All unused PC bags in blood banks are discarded after five days of storage (14). This imposes a great expenditure on the country heath system and creates a shortage of PCs for needy patients. It is of great importance to seek a solution. Platelet quality is affected by certain factors, such as the method of preparation, storage media, storage container and donor characteristics (38). It is a good idea to use media for storage of platelets that can increase the shelf-life of cells with better cellular metabolism. Many studies have compared different PASs and their benefits (18-23, 38). In the current study, we chose the Composol PS solution as an adequate PAS for longterm platelet storage. Advantages of Composol as PAS against plasma include better functional and biochemical parameters, particularly glucose consumption, which improves cellular metabolism and more platelet viability and increased life span. In addition, the use of PASs can saves more plasma for use in other situations, less plasma exposure of PC recipients, and decreased adverse reactions to transfusions (18).

To date, no previous study reported the expression pattern of platelet miRNAs in PCs stored in PAS. The present study was the first that compared the expression of a platelet miRNA (miR-16) in Composol and plasma media. For the comparison between the two PC groups, we replaced the plasma with Composol under sterile conditions. When using PAS, the usual mixture composition is 20-50% plasma and 50-80% PAS (38). In our study, the final mixture in the Composol samples was 30% plasma and 70% Composol solution.

Furthermore, to ensure that adequate platelets existed before the beginning of the miRNA extraction, an original bag with less than 1×10^12^ platelets/L on the preparation day (day zero) was rejected. In addition, centrifugation of PC samples at 96 g for 4 minutes effectively reduced the number of white blood cells and other non-platelet components, and had a minimal effect on reducing platelet counts. We sampled both PC groups simultaneously on days 1, 3, 5 and 7 of storage.

Results of the expression pattern of miR-16 in qRT-PCR cyclers gave raw CT results for each test reaction. However, raw CT results are not reliable and are not good parameters to show the changes in gene expression. We used REST software to analyse the primary results as fold changes according to the comparative CT method. This method is a relative quantification of gene expression in which the amount of target gene is calculated by relative expression of a reference housekeeping gene and is equal to 2^-∆∆CT^. The ∆CT implies the difference between CTs of the target and reference genes, and ∆∆CT is the difference in two ∆CTs such as between the test and calibrator or treated and untreated samples (24). In our study, the ∆∆CT was the difference between the ∆CTs of the Composol (test) group and plasma (control) group samples.* RNU6* was used as the endogenous reference and internal control gene for miR-16 expression normalization for all storage time points. So, the given fold change meant the fold increase or decrease in the expression of the target gene compared to our reference gene. Results of real-time analysis showed an obvious increase in miR-16 expression during storage in both group samples. However, this increase was slower in the Composol group. Furthermore, miR-16 expression was clearly down-regulated in the Composol group for all of the assessed storage days, in comparison to plasma group in corresponding days. Hence, miR-16 is a factor of apoptosis. This finding is of great importance and confirms the results of other studies where PASs, including Composol PS, had positive effects on platelet metabolism and increasing the shelf-life of platelets in storage. However, the previous studies did not compare miRNA expression patterns in plasma and PASs media.

The WST-1 test was used for analysis of platelet viability during PC storage. Viable platelets produce NADH that causes a reduction of cell-impermeable and colourless tetrazolium salt to purple and soluble formazan dye at the cell surface. The greater amount of formazan dye formation indicates a greater number of active and living platelets (39). Our results of WST-1 test support the above mentioned finding about miR-16. The platelets showed more viability in Composol medium than in plasma. This means that the Composol group PCs had more viable platelets than control plasma PCs. Hence, this supports the finding that Composol can be a useful medium for long-term platelet storage.

## Conclusion

The present study aimed to determine the effect of a PAS, Composol, on miR-16 expression during platelet storage. miR-16 had an increased expression pattern in all PCs in the control (plasma) and test (Composol) groups during storage. Furthermore, we showed that miR-16 expression decreased effectively in PCs stored in the Composol medium. We concluded that Composol might be a good choice as a PAS for longterm storage of platelets because it has the potential to elevate the shelf-life of platelets stored at 22˚C. Additional, in-depth with more samples integrated are needed to clearly confirm the above results. Analyses of other platelet miRNAs during PC storage would also be important.
